# Do Decision Support Tools Decrease the Prevalence of Hospital-Acquired Venous Thromboembolisms When Compared to Clinical Judgement? A Single-Center Pre–Post Study

**DOI:** 10.3390/jcm13133854

**Published:** 2024-06-30

**Authors:** Mohammad Abdulelah, Omar Haider, Matthew McAuliffe, Leen Al-Faris, Jasmine Paadam, Venkatrao Medarametla, Reva Kleppel, Kirti Joshi

**Affiliations:** Department of Internal Medicine, University of Massachusetts Chan Medical School—Baystate Regional Campus, Springfield, MA 01199, USAreva.kleppel@baystatehealth.org (R.K.);

**Keywords:** decision support tool, hospital-acquired venous thromboembolisms, risk assessment model

## Abstract

**Introduction:** Hospital-acquired venous thromboembolisms (HA-VTEs) carry a significant health burden on patients and a financial burden on hospitals due to reimbursement penalties. VTE prophylaxis at our institute was performed through utilizing an order set based on healthcare professionals’ perceived level of risk. However, the use of standardized risk assessment models is recommended by multiple professional societies. Furthermore, integrating decision support tools (DST) based on the standardized risk assessment models has been shown to increase the administration of appropriate deep vein thrombosis (DVT) prophylaxis. Nonetheless, such scoring systems are not inherently flawless and their integration into EMR as a mandatory step can come at the risk of healthcare professional fatigue and burnout. We conducted a study to evaluate the incidence of HA-VTE and length of stay pre- and post implementation of a DST. **Methods:** We conducted a retrospective, pre–post-implementation observational study at a tertiary medical center after implementing a mandatory DST. The DST used Padua scores for medical patients and Caprini scores for surgical patients. Patients were identified through ICD-10 codes and outcomes were collected from electronic charts. Healthcare professionals were surveyed through an anonymous survey and stored securely. Statistical analysis was conducted by using R (version 3.4.3). **Results:** A total of 343 patients developed HA-VTE during the study period. Of these, 170 patients developed HA-VTE in the 9 months following the implementation of the DST, while 173 patients were identified in the 9 months preceding the implementation. There was no statistically significant difference in mean HA-VTE/1000 discharge/month pre- and post implementation (4.4 (SD 1.6) compared to 4.6 (SD 1.2), confidence interval [CI] −1.6 to 1.2, *p* = 0.8). The DST was used in 73% of all HA-VTE cases over the first 6 months of implementation. The hospital length of stay (LOS) was 14.2 (SD 1.9) days prior to implementation and 14.1 (SD 1.6) days afterwards. No statistically significant change in readmission rates was noted (8.8% (SD 2.6) prior to implementation and 15.53% (SD 9.6) afterwards, CI −14.27 to 0.74, *p* = 0.07). Of the 56 healthcare professionals who answered the survey, 84% (*n* = 47) reported to be dissatisfied or extremely dissatisfied with the DST, while 91% (*n* = 51) reported that it slowed them down. **Conclusions:** There were no apparent changes in the prevalence of HA-VTE, length of stay, or readmission rates when VTE prophylaxis was mandated through DST compared to a prior model which used order sets based on perceived risk. Further studies are needed to further evaluate the current risk assessment models and improve healthcare professionals’ satisfaction with DST.

## 1. Introduction

Hospital-acquired venous thromboembolism (HA-VTE) is a potentially preventable, yet life-threatening condition characterized by significant morbidity and mortality rates [1]. It is projected that over 600,000 symptomatic VTE events occur annually in the United States, many of which are hospital-acquired [2]. Furthermore, such events place significant burdens on healthcare systems, in terms of financial costs, a prolonged length of stay (LOS), and high readmission rates [3,4,5]. The financial costs have a large financial impact upon healthcare systems [6]. Additionally, hospitals are negatively impacted when hospitalized patients develop HA-VTE due to previously set policies. For instance, governmental organizations such as the Centers for Medicare and Medicaid Services, as well as multiple state-based cost review commissions, enforce monetary penalties for HA-VTE [7]. It is pertinent to note that not all HA-VTE events are preventable [8]. Thus, The International Society on Thrombosis and Haemostasis and the American Heart Association (AHA) emphasize standardized VTE risk assessment rather than an institution’s VTE rate as a marker of quality and affecting care [9,10].

Standardized risk assessment models (RAMs) for HA-VTE have been an area of active research. Multiple models for standardized RAMs have been developed, such as the Padua medical RAM and Caprini surgical RAM [11], while other groups have incorporated the testing of D-Dimer into clinical RAMs with an observed improvement in risk stratification [12]. Often, these RAMs are incorporated into electronic medical records (EMRs) as pop-up reminders or decision support tools (DSTs) as an aid to clinicians to ensure appropriate prophylaxis is ordered given that 40% of VTE events in at-risk hospitalized patients could be prevented if established guidelines were adhered to [10,13,14,15].

A RAM calculates clinical risk factors that have been linked with a higher risk for developing HA-VTE. On the one hand, the Padua score calculates active cancer, reduced mobility, previous VTE, known thrombophilic conditions, recent trauma or surgeries, elderly age, cardiac or respiratory failure, myocardial infarction or ischemic stroke, acute infection or rheumatological disease, and obesity (BMI 30 kg/m^2^ or greater) with scoring points of 1, 2, and 3 for each variable and categorizes patients into low risk (<4) and high risk (≥4) [11]. On the other hand, the Caprini score accounts for age, biological sex, type of surgery, venous disease or clotting disorder, immobility, history of chronic obstructive pulmonary disease, myocardial infarction, inflammatory bowel disease, malignancy, obesity, and recent (<1 month) events such as sepsis, major surgery, pneumonia, congestive heart failure exacerbation, immobilizing plaster cast, lower extremity fracture, stroke, multiple trauma, or spinal cord injury causing paralysis [16]. The score also divides patients into different risk groups.

The use of RAM and DSTs has been studied by multiple groups with the aim of decreasing HA-VTE events. For instance, a study performed at a 900-bed teaching hospital showed a yearly incidence of 217 HA-VTEs, which dropped to 169 yearly events after implementing a RAM [17]. DSTs have shown a possible beneficial role in terms of improving adherence to guideline-directed prophylaxis [18]. A decrement in HA-VTE is of significant relevance and importance to hospital leaderships as an analysis of venous thromboembolism costs of illness in Europe revealed savings of up to EUR 7.3 billion per year if hospitals had utilized the appropriate preventive measures in place [6]. However, it must be noted that in the current era, clinicians are more aware of hospital-acquired complications such as VTE and the importance of risk stratification and prophylaxis [19,20,21,22].

EMR order sets have also been commonly used to ease physicians’ workflow [23]. They include a variety of common diagnostic tests and therapeutic medications for varying diseases. Utilizing order sets for HA-VTE has been previously linked with improvements in overall HA-VTE prophylaxis [24]. At our institute, we had an admission order set named “DVT prophylaxis” which divided patients at low, medium, and high risk of developing HA-VTE based on clinical judgment and perceived risk. Based on the risk group suspected, the order set had suggestions for prophylaxis according to the ninth edition of the American College of Chest Physicians (ACCP) Evidence-Based Clinical Practice Guidelines. The options available were no prophylaxis, sequential compression devices, enoxaparin, and subcutaneous heparin [25]. Utilizing such order sets led to a subjective assessment of risk level with variety between healthcare professionals.

We conducted this study to determine whether the prevalence of HA-VTE decreases once a DST is utilized in comparison to our old model which was based on clinical judgment. We also planned to evaluate possible changes in healthcare utilization in terms of hospital LOS and readmission rates.

## 2. Methods

We conducted this retrospective observational study at a single academic tertiary care referral center. This pre–post-implementation study was designed to evaluate the outcomes of implementing a DST for prophylaxis against HA-VTE in terms of the incidence of HA-VTE and hospital length of stay. The DST was implemented on 1 June 2023 with the aim of aiding with risk assessment and the prescription of VTE prophylaxis. This study was conducted over a period of 9 months after implementation (1 June 2023–29 February 2024). Data from the nine months preceding the implementation (1 September 2022 to 31 May 2023) were also collected for comparison.

This study followed the Strengthening the Reporting of Observational Studies in Epidemiology (STROBE) guidelines for descriptive observational studies [26]. This project was reviewed by our hospital’s Institutional Review Board (IRB) and an exemption from review was granted due to the nature of this observational study. None of the authors have any applicable competing interests.

- Patient cohort

The hospital’s data analysis team identified and reported all patients who were discharged from the hospital during the study period. No exclusions were applied. Thereafter, patients who developed a hospital-acquired VTE were identified by using the 10th revision of the International Statistical Classification of Diseases and Related Health Problems (ICD-10) codes I26, I269, I260, I801, I802, I803, I82, I822, and I829. All patients who developed HA-VTE were reported with no exclusions. Once a secure and encrypted database including patients’ confidential medical record numbers and specific encounter identifying numbers was created, medical residents accessed patients’ charts and confirmed the timing of VTE in relation to hospitalization through reviewing imaging reports as documented by radiology consultants. Timing was defined as within hospitalization or within 90 days of hospital admission [3].

- DST design

The DST was in the form of a pop-up alert, encountered upon accessing patients’ charts. Notably, pop-ups prompting the ordering of the DST would be displayed every time the patient’s chart was accessed if the DST had not been ordered. The DST utilized the Padua risk score for medical patients and the Caprini risk score for surgical patients, both of which were automatically derived from EMRs once an admission order was placed. These risk assessment models were selected as they have been previously validated and recommended by professional societies [27,28,29]. The DST would then display recommendations based on the risk group level per the ACCP recommendations [25]. Healthcare professionals would then be obligated to select one of the recommended forms of chemical or mechanical thromboprophylaxis methods, and an option to defer thromboprophylaxis was available but a reason for deferring must have been manually documented. Order sets, which were utilized prior to the inception of the DST, were hidden from the system to increase compliance with the VTE Advisor. Free text orders for DVT prophylaxis were also removed from the system. Prior to implementation of the DST, education was provided to all healthcare professionals in terms of demonstrative lectures and detailed e-mails. If the DST was not ordered, a pop-up alert referring the healthcare professional to order the DST would appear whenever the patient’s chart was opened.

- Outcomes evaluated

This study analyzed multiple trends related to HA-VTE and utilized the DST. Our project’s main aim was to compare the incidence of HA-VTE before and after the implementation of the DST. The secondary outcomes evaluated included healthcare burdens in terms of LOS and readmission rates as well as compliance with ordering the DST.

- Data collection

Utilizing electronic medical records and the hospital’s data analysis team’s records for inpatient discharges, the incidence of HA-VTE, length of stay, and readmission rates were collected. Other collected variables were compliance with ordering the DST and initial VTE presentation (upper extremity, lower extremity, or pulmonary embolus); however, these were limited to the first 6 months after implementation. Data were stored in an encrypted cloud with access limited to the authors of this study. Patient identifiers were removed prior to analysis to protect patient confidentiality.

- Survey

A 6-question survey was also sent to healthcare professionals practicing at our hospital. The survey was anonymous to better understand healthcare professionals’ perspective regarding the implementation of a DST. The survey was sent to a total of 167 eligible healthcare professionals by the primary investigator of our study. The survey was sent to healthcare professionals (attendings and residents) who have been in practice throughout the 18 months of this study (September 2022 to February 2024). The survey questions were as Appendix A.

The survey answers were securely stored. Access to survey results was limited to authors of this study. A total of 56 healthcare professionals answered the survey.

- Statistical analysis

Statistical analysis was conducted using R (version 3.4.3). Descriptive statistics were carried out to offer a snapshot of essential features within the dataset. Categorical variables are presented as absolute numbers and percentages of data entries. Data were tested for normality through Kolmogorov–Smirnov and confirmed through theoretical quantile (QQ) plotting. Normally distributed metric variables, such as incidence rates, are presented as means and standard deviations, while non-normally distributed data, such as length of stay, are presented as medians and interquartile ranges. Comparative analysis was performed to understand differences in subgroups. Continuous parametric variables were compared using the independent-sample t test while non-parametric variables were compared using the independent-sample Mann–Whitney-U test. 

The confidence interval was set to 95% and the margin of error accepted was set to 5%. Therefore, the following *p*-values were considered significant:

- *p* > 0.05: statistically insignificant;
- *p* < 0.05: statistically significant.

## 3. Results

A total of 343 cases of HA-VTE were identified over the 18-month period of this study. A total of 173 cases were diagnosed in the first 9 months prior to implementing the DST and 170 were diagnosed afterwards. Data from the initial 6 months of implementation of the DST revealed that the majority (38%, *n* = 40) of cases affected the lower extremity while 29% (*n* = 31) affected the upper extremities. A proportion of 20% (*n* = 21) were first identified as pulmonary emboli. The location was unspecified in 13% of cases (*n* = 14).

**Figure 1:**
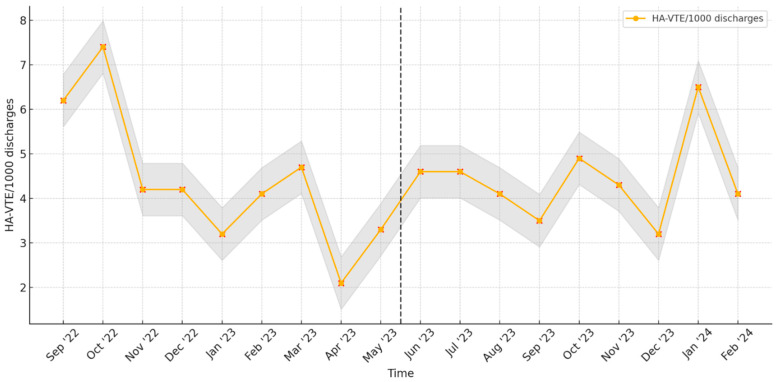
Monthly variation in incidence of hospital-acquired VTEs per 1000 discharges is repressented by orange dots. The DST was first implemented in June 2023. The dotted line indicates the implementation of DST while the shaded area (gray) represents the monthly 95% confidence interval for the incidence.

Pre-implementation, the mean incidence rate of HA-VTE events was 4.4 (SD 1.6) cases/1000 discharges/month. Once the DST was implemented, we noted a mean of 4.6 (SD 1.2) cases of HA-VTE/1000 discharges/month (Table 1). There was no statistically significant difference in the incidence of mean HA-VTE events/1000 discharges with the implementation of the DST (confidence interval [CI] −1.6 to 1.2, *p* = 0.8). The DST was used in 73% of all HA-VTE cases over the first 6 months of implementation. There were no temporal changes in compliance over the evaluated period. Figure 1 shows the monthly variation in HA-VTE events.

The median LOS for those who developed HA-VTE was 15 (IQR 17) days over the study period; on the other hand, the average LOS at our hospital for those without HA-VTE over the same period was 4 (IQR 4) days. This suggests a statistically significant difference in LOS (mean rank of 48,849 and 27,452 respectively; U = 1,909,814; *p* < 0.001). 

Comparing the LOS for those with HA-VTE prior to and subsequent to the implementation of the DST revealed a median LOS of 14 (IQR 18) and 15 (IQR 17) days, respectively (mean rank of 153 and 158, respectively; U = 11,647; *p* = 0.6) (Table 2). 

The mean 30-day readmission rate (30 day) in those who developed HA-VTE prior to using the DST was 8.8% (SD 2.6), while afterwards, it was 15.53% (SD 9.6) (95% CI −14.27 to 0.74, *p* = 0.07). 

The survey had a response rate of 34% (56 of 167). A proportion of 87.5% (*n* = 49) of healthcare professionals preferred ordering prophylaxis through an order-set over the new DST. Most physicians were either dissatisfied or extremely dissatisfied with implementing the DST. A proportion of 91% (*n* = 51) reported that the DST slowed them down. The remaining survey answers can be found in the online Supplementary Materials. 

## 4. Discussion

Our findings suggest that implementing a DST did not change the overall incidence of HA-VTE when compared to using a conventional order set. The prevalence of HA-VTE has been rising nationally with more pronounced adverse effects [3]. The ideal modality of VTE risk assessment has been an emerging area of study given the mixed and conflicting outcomes previously reported when different diagnostic and prophylaxis algorithms were followed [30]. For instance, electronic medical record reminders have been found to be beneficial in increasing the prescription of DVT prophylaxis [31,32]. Furthermore, DSTs incorporated into EMRs have also been shown to improve guideline-directed VTE risk assessment [32,33]. However, our institute’s experience with implementing a mandatory DST was different from prior published research. A recently published study by Spyropoulos et al. revealed that using DSTs increased appropriate thromboprophylaxis and reduced the overall incidence of HA-VTE [34]. However, the researchers used a risk assessment tool that incorporated clinical characteristics as well as D-Dimer testing, unlike our study which utilized the Padua and Caprini RAM that revolve around clinical characteristics only [11]. Therefore, this raises the question of whether our institute used the correct risk assessment tool and whether outcomes would have been different if D-Dimer was also evaluated [35]. Notably, the Padua score was found to be associated with a higher mortality risk but failed to show a positive correlation between the administration of anticoagulation and a reduced rate of VTE or mortality [36,37,38]. This was also the case with multiple other risk assessment models [39]. Thereafter, multiple groups are evaluating different electronic algorithms to enhance the predictability of HA-VTE rather than relying on clinical risk predicting scores [40]. Another possible reason for our findings is the prior availability of an intuitive order-set used to facilitate ordering prophylactic agents based on perceived risk, which was perhaps less time-consuming. Notably, utilizing order sets has been previously shown to improve VTE prophylaxis prescription patterns and the overall LOS [24]. However, our prior system depended on healthcare professionals’ perceived level of risk, and such a subjective inference of risk level has been reported to increase inappropriate VTE prophylaxis when compared to using standardized assessment models [41]. Regardless, with rapid advancements in technology, and the emergence of artificial intelligence in medicine, better risk assessment and prophylaxis might be soon incorporated in clinical practice [42]. Ultimately, a combination of algorithmic DSTs as well as maintaining a component of clinical judgment and intuition might lead to a more appropriate prophylaxis and overall lower HA-VTE given the current state of risk assessment models. Therefore, we raise the question of whether implementing a DST with the ability for clinicians to override recommendations would have yielded different results than what was observed in our cohort. Nonetheless, DSTs remain a cornerstone in the current era of medicine as they have been proven to improve outcomes in a plethora of clinical settings [42,43,44].

The incorporation of algorithmic DSTs into EMRs has been an ongoing trend over the past decade, aiming to improve clinicians’ workflow, increase compliance with societal recommendations, and avoid potential complications [45,46]. The observed compliance rate in our study aligns with some of the previously published literature [47]. Such findings indicate that measures taken such as the removal of previously existing order sets and the placement of the DST at critical clinical decision-making points (opening a chart or trying to place an order) optimized the likelihood that the DST was followed [10]. However, it is worth noting that alert fatigue associated with many DSTs may contribute to physician burnout given the interruptive and nonurgent nature of alerts as it might lead to disturbing clinicians’ thought processes and workflow [48]. Our study was concordant with such literature as the majority of healthcare professionals surveyed reported dissatisfaction with the DST and it slowed their workflow. As there is more integration of DSTs, further studies are needed to evaluate alert fatigue and compliance due to their potential of limiting the tool’s impact. Regardless, emphasis on the potential merits of the system and workflow integration has been shown to improve universal confidence and acceptance [49,50]. Thereafter, decision makers must weigh the presumed clinical risks and benefits of implementing such DSTs and compare them to the implementing costs, workload required, and healthcare professionals’ preferences. 

Our study reinforces the healthcare burden associated with HA-VTE. We noticed a significantly higher LOS when comparing those with HA-VTE to those without. Such findings are of significant importance as VTEs are known to cost the US healthcare system around 10 billion USD annually [51]. Additionally, HA-VTE has been reported to increase total encountered hospitalization costs; however, no recent data regarding direct hospital-afflicted costs and outcomes have been published [52,53]. Given the fact that HA-VTE has been reported to be the second most common complication to prolong the length of hospitalization, extensive economical evaluation of the burden associated with HA-VTE should be conducted [54]. This is financially relevant as Medicare implemented the Hospital-Acquired Condition Reduction Program, which decreases hospital reimbursement rates for hospital-acquired conditions such as VTE [55]. Thus, such financial challenges call for improving our risk assessment and prophylaxis patterns against HA-VTE to ensure patient safety and satisfactory reimbursement. Additionally, the AHA has published a proposed policy to decrease HA-VTE by 20% by the year 2030 [10]. Ultimately, multiple authors believe that the ideal state for HA-VTE prevention should include standardized risk assessment, the provision of risk-appropriate VTE prophylaxis, the prevention of missed chemoprophylaxis doses, and the definition and tracking of rates of preventable VTE [55].

Our study has multiple limitations. First, a major limitation of our single-center pre–post study is that a natural increase in HA-VTE prevalence at the same time as the DST was implemented cannot be excluded. We compared our findings to our hospital’s records for the 9 months prior to implementation to better understand trends. However, this approach assumes that the comparison reflects the natural heterogeneity in clinical complexity and trajectory of patients. Within the same context, the absence of a control group in our study design limits our ability to definitively attribute observed changes in HA-VTE prevalence to implementing the DST alone. Other external factors, such as temporal trends, that occurred concurrently with the intervention period may have influenced the outcomes. For instance, our study did not account for the seasonal variation in VTE incidence, as it was not conducted over a complete 12-month period [56]. Furthermore, the short follow-up period of our study indicates a possible variability in DST use during the immediate post-implementation period. We tried to overcome such bias by providing education and stressing the importance of using the DST during weekly meetings. Nonetheless, longer-term analysis is required to understand the trajectory in outcome trends based on the feedback provided and account for variables such as seasonality. Third, data regarding appropriate prescriptions were not available to our group. Fourth, our analysis was confined to a single academic medical center; thereafter, variability might be observed if DSTs are implemented in other practice settings. Lastly, our survey had a low response rate, and therefore, larger qualitative studies are needed to understand healthcare professionals’ satisfaction rates. 

## 5. Conclusions

Our findings did not suggest beneficial outcomes from the widespread implementation of a DST for HA-VTE prophylaxis in terms of HA-VTE incidence or hospital LOS compared to using admission order sets based on perceived clinical judgment. Additionally, high dissatisfaction was noted among surveyed healthcare professionals. Further studies are needed to evaluate appropriate risk assessment tools and their implementation into clinical practice with emphasis on improving patient safety and healthcare professionals’ workflow. 

## Figures and Tables

**Table 1:** Monthly incidence of HA-VTE as well as number of total hospital discharges. The incidence rate of HA-VTE was calculated as the incidence of HA-VTE per 1000 discharges per month. HA: hospital-acquired; VTE: venous thromboembolism.

Month	Cases	Discharges	Incidence per 1000 Discharges
September 2022	24	3897	6.2
October 2022	29	3933	7.4
November 2022	16	3772	4.2
December 2022	17	4082	4.2
January 2023	13	4078	3.2
February 2023	15	3689	4.1
March 2023	19	4085	4.7
April 2023	8	3808	2.1
May 2023	13	3931	3.3
Implementation of decision support tool			
June 2023	19	4097	4.6
July 2023	19	4141	4.6
August 2023	17	4148	4.1
September 2023	14	3977	3.5
October 2023	20	4045	4.9
November 2023	17	3955	4.3
December 2023	13	4067	3.2
January 2024	31	4802	6.5
February 2024	18	4417	4.1

**Table 2:** The median length of stay for all patients admitted to the hospital as well as those with hospital-acquired venous thromboembolisms over an 18-month period spanning September 2022 to February 2024. HA: Hospital acquired; VTE: venous thromboembolism; LOS: length of stay.

Month	All-Cause Median LOS (IQR)	HA-VTE Median LOS (IQR)
September 2022	4 (5)	14 (10)
October 2022	4 (5)	14 (17)
November 2022	4 (5)	12 (9)
December 2022	4 (4)	18 (23)
January 2023	4 (6)	14 (23)
February 2023	5 (5)	17 (16)
March 2023	4 (6)	15 (12)
April 2023	4 (4)	12 (11)
May 2023	4 (5)	26 (20)
Implementation of decision support tool		
June 2023	4 (4)	13 (11)
July 2023	4 (3)	18 (26)
August 2023	4 (5)	16 (26)
September 2023	4 (5)	15 (17)
October 2023	4 (4)	14 (8)
November 2023	4 (5)	15 (25)
December 2023	4 (5)	15 (7)
January 2024	4 (5)	19 (25)
February 2024	4 (4)	18 (24)

DST: decision support tools; VTE: venous thromboembolisms.

## Data Availability

The raw data supporting the conclusions of this article will be made available by the authors on request.

## Supplementary Materials

The following supporting information can be downloaded at: https://www.mdpi.com/article/10.3390/jcm13133854/s1, Figure S1: Degree of healthcare proffessional’s satisfaction with the DST based on an anonymous survey sent to healthcare proffesionals at our hospital.

## Appendix A

**Question**	**Answers**
How satisfied are you with the new DST?	○ Extremely dissatisfied ○ Dissatisfied ○ Neutral ○ Satisfied ○ Extremely satisfied
How has the DST changed your workflow?	○ Slowed me down ○ Did not affect my workflow ○ Made me faster
Has it changed your prescription patterns?	○ Made me prescribe more prophylaxis ○ Did not change my prophylaxis patterns ○ Made me prescribe less prophylaxis
Do you think it reduced the overall incidence of VTE events?	○ Yes ○ No ○ Unsure
Do you think it reduces overall VTE associated outcomes?	○ Yes ○ No ○ Unsure
Which system do you prefer?	○ Old system (Order set based) ○ New system (DST Based)
DST: decision support tools; VTE: venous thromboembolisms.	
