# Supplementary material for: Do Decision Support Tools Decrease the Prevalence of Hospital-Acquired Venous Thromboembolisms When Compared to Clinical Judgement? A Single-Center Pre–Post Study
Source: J Clin Med. 2024 Jun 30;13(13):3854. doi: 10.3390/jcm13133854 (PMC11242558; doi:10.3390/jcm13133854)
Supplement: Supplementary file 1 [file jcm-13-03854-s001.zip › jcm-3046050-supplementary.pdf]

Supplementary Materials

The survey also revealed that 68% (n=38) did not report a change in prophylaxis prescription frequency, while 32% (n=18) reported that they were prescribing less prophylaxis after the implementation of the DST. 57% (n=32) of the physician's surveyed had the impression that the DST did not decrease the frequency of HA-VTE, while 38% (n=21) were unsure regarding any changes in the frequency of HA-VTE. On the other hand, 55% (n=31) did not think that implementing the DST lead to any changes in overall VTE associated outcomes, while 38% (n=21) were unsure.

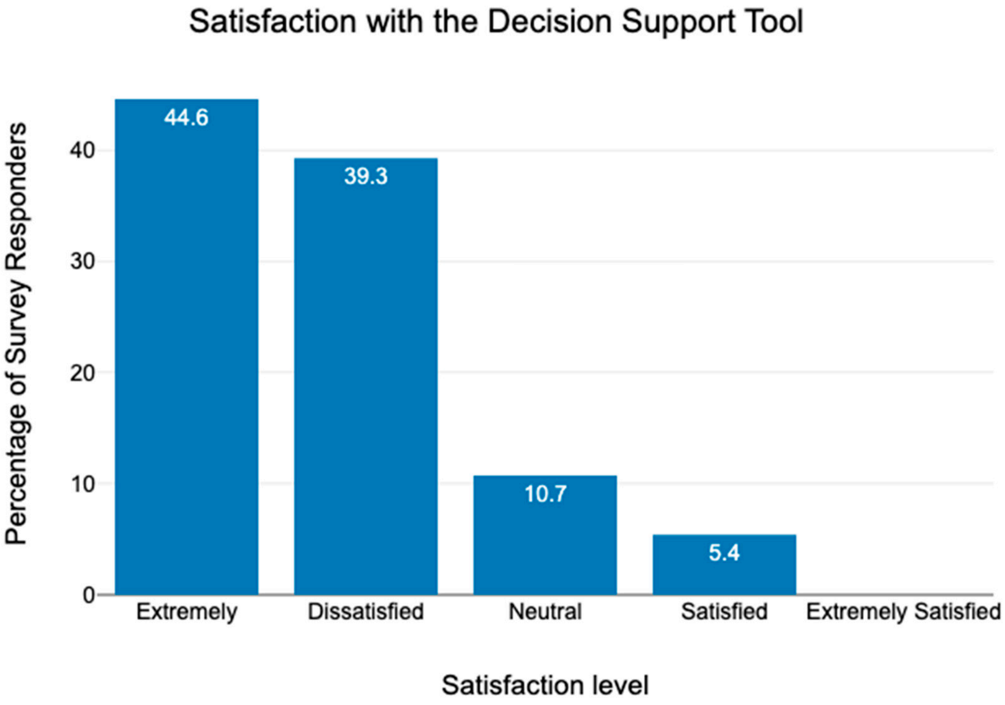

Figure S1. Degree of healthcare proffessional's satisfaction with the DST based on an anonymous survey sent to healthcare proffesionals at our hospital. The survey was sent to 167 healthcare proffesionals. The choices were extremely dissatisfied, neutral, satisfied and exremely satisfied.
